# Development of Novel Blown Shrink Films from Poly(Lactide)/Poly(Butylene-Adipate-co-Terephthalate) Blends for Sustainable Food Packaging Applications

**DOI:** 10.3390/polym14142759

**Published:** 2022-07-06

**Authors:** Arianna Pietrosanto, Annalisa Apicella, Paola Scarfato, Loredana Incarnato, Luciano Di Maio

**Affiliations:** Department of Industrial Engineering, University of Salerno, Via Giovanni Paolo II 132, 84084 Fisciano, SA, Italy; arpietrosanto@unisa.it (A.P.); anapicella@unisa.it (A.A.); lincarnato@unisa.it (L.I.); ldimaio@unisa.it (L.D.M.)

**Keywords:** shrink film, extrusion blowing, biopolymer, PLA/PBAT, sustainable packaging

## Abstract

Heat-shrinkable films, largely made of polyolefins and widely employed in the packaging sector as collation or barrier films, due to their short service life, are held responsible for high environmental impact. One possible strategy for reduction in their carbon footprint can be the use of biodegradable polymers. Thus, this work aimed to develop novel, heat-shrinkable, fully biodegradable films for green packaging applications and to analyze their functional performance. Films were obtained from blends of amorphous polylactic acid (PLA) and poly(butylene-adipate-co-terephthalate) (PBAT) at different mass ratios and compatibilized with a chain extender. They were produced by means of a lab-scale film blowing extrusion apparatus and characterized in terms of physical–mechanical properties and shrinkability. The influence of the processing parameters during the extrusion blowing process on the films’ behavior was investigated, highlighting the effects of blend composition and stretching drawing conditions. Shrinkage tests demonstrated that the produced films have shrinkability values in the typical range of mono-oriented films (ca. 60–80% in machine direction and ca. 10–20% in transverse direction). Moreover, the shrinkage in machine direction increases both with the mass flow rate, the take-up ratio to blow-up ratio and the bubble cooling of the film blowing process, and with the PLA content into the blend. In particular, films at higher PLA content also exhibit higher transparency and stiffness.

## 1. Introduction

Heat-shrinkable films are widely employed for many packaging applications [1]. These kinds of films are characterized by the capacity to considerably reduce their dimensions when exposed to heat, allowing them to tightly wrap the products they are enveloping.

Conventional methods to obtain a heat-shrinkable film consist of a two-step procedure: (i) stretching of the polymeric film at a temperature close to its softening point (that is slightly above the glass transition temperature of the amorphous phase of the polymer), to obtain molecular orientations in its amorphous phase; (ii) freezing the film in the oriented state. These orientations are then lost if the temperature is raised again by exposing the film to heat. The oriented polymeric segments turn back to their equilibrium position in the random coil state with the lowest entropic energy [2]. This process has the macroscopic effect of film shrinkage or, if the film is detained, of tension raising within the film itself.

The film blowing extrusion process represents the most widespread process on an industrial scale to manufacture shrink films without the added expense and complexity of other orientation techniques such as tenter frame or double bubble systems [3]. In the film blowing process, the macromolecules can be oriented in two directions: machine direction (MD) and transverse direction (TD). Depending on the entity of MD and TD orientation, shrink films are divided in two categories: mono-oriented films (MD shrinkage between 60–80%, TD shrinkage between 10–20%) and bi-oriented films (MD shrinkage between 50–60%, TD shrinkage between 30–40%) [4]. A further classification is based on the film thickness: thin shrink films have a thickness up to 50 μm and heavy-duty shrink film which have a thickness ranging from 50 to 150 μm [4].

In the film blowing process, the orientation related to film stretching is achieved by adjusting the processing parameters which can be summarized as follows: take up ratio (TUR), blow up ratio (BUR), mass flow rate, temperature profile and cooling conditions. In particular, the MD orientation is related to the TUR and accomplished by the tuning of the haul-off speed of the polymer exiting from the die. On the other hand, TD orientation is achieved through the inflation of air, which causes the increase in the bubble diameter and the stretching of the polymer mainly in this direction. This orientation is related to the BUR [5]. It is worthy to note that polymer orientation in MD and TD occurs below the frost line height, between the glass transition and the melting temperature of the polymer. Since this condition strongly depends on the above-mentioned processing parameters, the desired shrinkage of the film can be achieved only if a suitable balance the film blowing process conditions is accomplished.

From a literature survey, various studies were performed to correlate the extrusion blowing parameters with the film shrinkage. Patel et al. [6] reported that MD shrinkage of the linear low-density polyethylene (LLDPE) blown films was better correlated to TUR/BUR than TUR, because the MD shrinkage is also affected by the orientations made in TD. Menges et al. [7] found that for low-density polyethylene (LDPE) blown film, changing the die geometry can allow us to obtain balanced shrinkage in MD and TD and the increase in the frost line height can led to an increase the shrinkage in the TD, with a very low influence in the MD. Luo et al. [5] prepared a polyethylene (PE) three-layer film using a film blowing plant equipped with a rotating die, and they found that the mandrel rotation speed significantly improved the shrinkage of the film in the TD rather than BUR. Moreover, Torres et al. [8] developed a tool to predict the extrusion blowing processing, mechanical and shrink properties of PE blends.

Currently, most of the published papers regarding polymer orientation by film blowing technique are focused on PE or on polyolefins, since they are commonly employed for this application, holding more than 45% of the global shrink films market [9]. The environmental problems related to the disposal of such polymers, is pushing the research towards the development of eco-compatible solutions, aimed at improving the environmental sustainability of packaging materials. In this context, the use of biodegradable polymers as alternative materials for these kinds of products can be considered as an urging feature [10].

Among the biodegradable polymers, polylactic acid (PLA) is one of the most used, thanks to its good extrusion processability, high stiffness and transparency, high aroma barrier and suitability for direct food contact applications [11,12,13]. However, PLA is also characterized by brittleness and low melt strength. This latter feature, in particular, limits its processability window for blowing extrusion, inhibiting the use of high stretching ratios that are necessary for obtaining shrinkable films. Therefore, one of the current strategies to overcome these drawbacks is the combination of PLA, by blending or lamination, with other biodegradable polymers having more suitable rheological behavior and better mechanical properties [2,14,15,16,17,18,19]. Promising results were obtained by the blending with poly(butylene-adipate-co-terephthalate) (PBAT), an aliphatic aromatic copolyester characterized by a good processability and mechanical properties quite similar to polyethylene, i.e., low elastic modulus and high ductility, [19,20]. Although PBAT is inherently not compatible with PLA, the two resins can be effectively compatibilized in situ by reactive extrusion, obtaining blends more suitable for the film blowing process [21,22,23,24,25,26,27]. However, despite the numerous studies focused on production and characterization of blown films from PLA/PBAT blends, there is very little information on their use for development of shrink films by film blowing, and only reported in patent literature [28,29], therefore further investigations are needed.

Within our research activities aimed to design and develop innovative sustainable packaging solutions for application in the food sector, we performed a wide investigation focused on the relationships among composition, compatibilization by reactive blending, rheological behavior, processability by film blowing, and physical–mechanical properties of PLA/PBAT blends [13,17,23]. In this work, we intended to develop biodegradable shrink films for food packaging using PLA/PBAT compatibilized blends, and to investigate the effects of the processing parameters on the shrinkability of the films. PLA/PBAT films at two blend compositions were produced by a lab-scale film blowing plant at different processing conditions (i.e., TUR/BUR ratio, mass flow rate, and cooling speed), and then characterized in terms of shrinkability, morphology, transparency and mechanical properties, to highlight the effects of the extrusion blowing parameters on the film performance.

## 2. Materials and Methods

### 2.1. Materials

PLA 4060D, obtained from NatureWorksTM (Plymouth, MN, USA), has a content of D-isomer equal to 10 wt %, a specific gravity of 1.24 g/cm^3^ and a glass transition temperature between 55–60 °C. Ecoworld PBAT 009 was manufactured by Jin Hui Zhaolong (Lyuliang, China); it is constituted by the 29% of adipic acid, 26% of terephthalic acid and 45% of 1,4-butanediol, and it has a density of 1.26 g/cm^3^ and a melting temperature around 110–120 °C. Both PLA 4060D and Ecoworld PBAT 009 comply with EU and USA regulations for direct food contact applications. A chain extender named as Joncryl ADR-4368C was supplied by BASF (Ludwigshafen, Germany).

### 2.2. Production of the Films

PLA and PBAT pellets were dried under vacuum at 70 °C for 16 h prior to processing. Two different blends at PLA/PBAT mass ratios of 60/40 and 40/60, with 1 wt % of Joncryl (with respect to the total polymer content) as compatibilizer, were prepared and they are named as PLA60 and PLA40, respectively. The dry blends were melt-blended in a Collin ZK25 co-rotating twin extruder (D = 25 mm, L/D = 42) at a screw speed of 100 rpm with a mass flow rate fixed at approximately 0.9 g/s. A round die was used to produce a strand which was cooled by means of a water bath and pelletized.

Blown films were produced using a single screw extruder GIMAC (D = 12 mm, L/D = 24) with a temperature profile ranging from 180 °C to 130 °C from the hopper to the die. The blown film die, with a radial spiral mandrel distributor, is characterized by an inner diameter of 30 mm and a die gap of 0.8 mm. A take-up system (Teach-Line from Collin) was used to blow and draw down the extruded films, at different combinations of take up ratio (TUR) and blow up ratio (BUR). The TUR was changed by varying the mass flow rate and the nip rolls speed, while the BUR was changed by varying the quantity of inflated air.

BUR was defined as the ratio between the Bubble Diameter (*D_b_*) and the Die inner Diameter (*D_d_*), i.e.,

(1) $$\mathrm{BUR}=D_{b}/D_{d}$$

where *D_d_* was equal to 30 mm and *D_b_* was calculated as follows:

(2) $$D_{b}=2\times Layflat\;Width/\pi$$

TUR was calculated with the following relationship:

(3) TUR=Die Gap/(Film Thickness ×BUR)

where the Die gap was equal to 0.8 mm.

Films were produced at different combinations of cooling conditions, TUR, BUR and mass flow rate. Screw speed was used to tune mass flow rate while two cooling conditions were used by keeping open or closed the cooling air ring. 

Sample nomenclature is PLA X_Y, where X represents the relative content of PLA respect to PBAT in the blend, while Y is a progressive number that is linked to the different film blowing process conditions. The presence of an asterisk means that the film was produced without cooling air.

All the produced films and the respective process conditions are reported in Table 1 and Table 2 for PLA40 and PLA60 blend, respectively. Film thickness was determined in three different point over the width of the film with a maximum standard deviation of 0.7 µm.

### 2.3. Film Characterization

The shrinkage in MD and TD directions was determined according to the ASTM D-2732 standard using distilled water at 85 °C. Five replicates of each film were tested. The value % Shrinkage was calculated as follows:

(4) $$\%\;Shrinkage=\left(L_{0}-L_{f}\right)/L_{0}\times 100$$

where *L*_0_ is the initial length in the machine or transverse direction, and *L_f_* is length after shrinking in the MD or TD.

The thermal analysis was carried out using a Differential Scanning Calorimeter (DSC mod. 822, Mettler Toledo S.p.A., Milan, Italy) under a nitrogen flow (100 mL/min), to minimize thermo-oxidative degradation phenomena. Three scans were performed; samples were heated from −70 to 200 °C with a speed of 10 °C/min and held at 200 °C for 5 min. They were then cooled at −70 at 10 °C/min and heated again to 200 °C at 10 °C/min. The crystallinity degree of PBAT, *X_c_*, was calculated as follows:

(5) $$X_{c}=[\left({\Delta H}_{m}-{\Delta H}_{cc}\right)/\left(\Delta H_{m0}\times \varphi_{i}\right)]\times 100$$

where Δ*H_m_* and Δ*H_cc_* (J/g) are the PBAT’s heat of melting and heat of cold crystallization, respectively, Δ*H_m_*_0_ is equal to 114 [17] and *φ_i_* is the relative weight fraction of PBAT in the blend.

For the transparency tests, the films were cut into rectangular shapes and placed on the internal side of a spectrophotometer cell. The transmittance of the films was evaluated using a UV–Vis spectrophotometer (Lambda 800, Perkin Elmer Italia S.p.A., Milano, Italy) in the 800–200 nm region. The transparency of the films was measured at 560 nm. Five replicates of each film were tested. The percent transparency (*TR*_560_) was calculated as follows:

(6) $$TR_{560}=T_{r}/T_{0}\times 100$$

where *T_r_* is the transmittance with the specimen in the beam and *T*_0_ is the transmittance with no specimen in the beam.

Tensile testing of blown films was performed by a SANS dynamometer (mod. CMT 6000 by MTS, Shenzhen, China) equipped with a 100 N load cell. Rectangular specimens (width = 12.7 mm and length = 30 mm) were cut by a die cutter. The crosshead speed was set according to ASTM D822 standard. Mechanical properties were evaluated in the machine direction (MD) and in the transverse direction (TD). Results are an average of at least ten specimens.

## 3. Results and Discussion

### 3.1. Shrink Properties

The effects of the film blowing processing parameters and of the blend composition on the percentage of shrinkage were investigated. The percentage of TD and MD shrinkage for each film is reported in Table 3 and Table 4 for PLA40 and PLA60 blend, respectively.

Most of the produced films have MD shrinkage in the range 60–80% and TD shrinkage in the range 10–20%, in the typical range of mono-oriented films [4], which was the objective of the present work. Therefore, the effect of the process parameters was focused on the MD shrinkage.

#### 3.1.1. Effect of the TUR/BUR Ratio, Mass Flow Rate, and blend Composition

**Figure 1:**
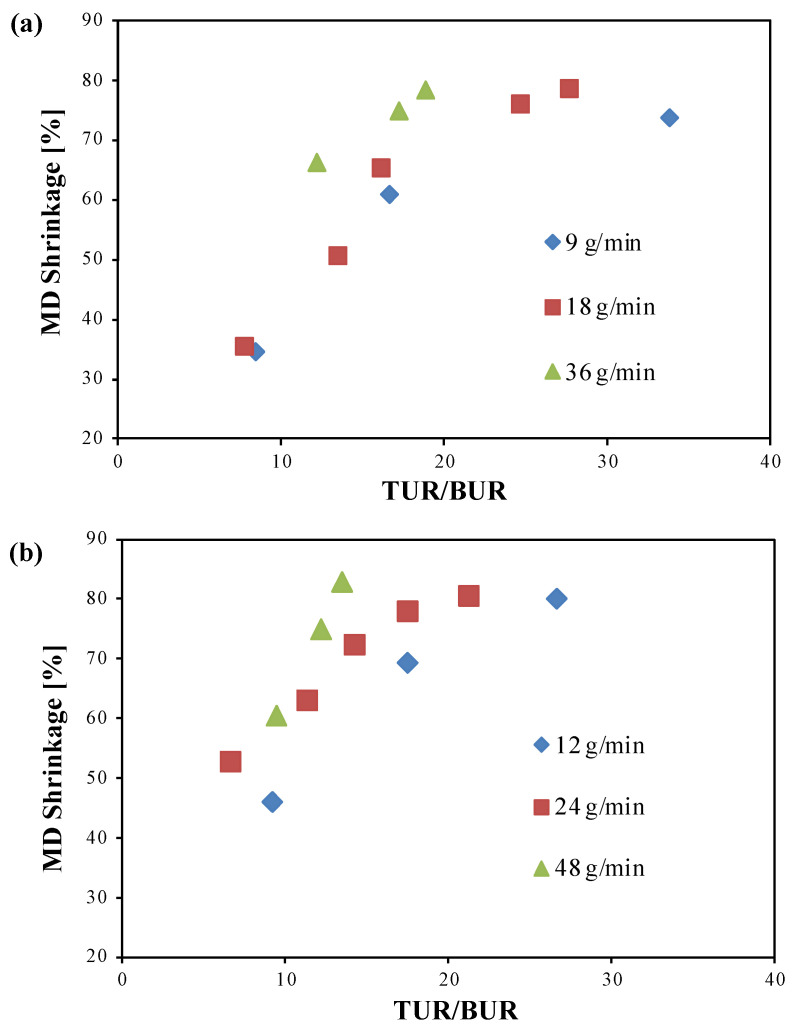
Percentage of shrinkage in MD versus TUR/BUR for (**a**) PLA40 and (**b**) PLA60 films, obtained with cooling air.

In Figure 1, the percentage of shrinkage in MD of the films obtained with cooling air are plotted versus the TUR/BUR ratio and as a function of the mass flow rate. The graphs show that for both the blends and for all the mass flow rates, the percentage of MD shrinkage increases with the increase in TUR/BUR ratio. In fact, at increasing TUR/BUR, which is achieved by the increase in the haul-off speed, polymer chains are stretched mostly in MD, therefore leading to higher MD shrinkage. The trend of the MD shrinkage as a function of the TUR/BUR ratio is almost linear. Then, for the lower mass flow rates, as the TUR/BUR increases, the slope decreases, and the MD shrinkage tends to a plateau value. This indicates the presence of a maximum TUR/BUR ratio, whose value depends on the mass flow rate and on the blend composition, after which there is no relevant effect on the MD shrinkage. The achievement of the plateau is attributable to the fact that, for high values of TUR/BUR, the macromolecules are already almost all oriented and a further increase in this ratio does not cause significant additional orientations and consequently increase in the percentage of shrinkage. However, for the highest mass flow rates tested, the plateau value is not detectable because high TUR/BUR ratios have not been achieved. Moreover, for all the mass flow rates tested, since the thickness of the film decreased with the increase in the TUR/BUR ratio due to higher increase in the MD stretching, the effect of the TUR/BUR ratio was enhanced. However, this effect is partly compensated by the fact that as the thickness decreases, the cooling of the film becomes faster. This involves a more rapid freezing and immobilization of the macromolecules, thus reducing the effect of MD stretching on their orientation.

MD shrinkage was affected by the mass flow rate too. At the same TUR/BUR, increasing the mass flow rate led to an increase in the MD shrinkage. The increase in the mass flow rate determines higher shear stress on the polymer melt passing through the die, leading to higher stretching of the macromolecules in the MD. Moreover, to maintain a constant TUR and TUR/BUR ratio, films produced with higher mass flow rates were obtained with a larger nip rolls rate, which further enhanced the orientation in MD.

The composition of the blends also contributes to the different behavior of films. Blend with a PLA content of 60% gave higher values of MD shrinkage compared to the blend with a PLA content of 40%. This is because the PLA used in this study is amorphous so, when it is heated above its glass transition temperature, it completely loses its orientations, leading to a higher shrinkage of the resulting film.

#### 3.1.2. Effect of the Bubble Cooling

The shrinkage data of the samples obtained with and without the application of cooling air are reported in Table 3 and Table 4.

**Figure 2:**
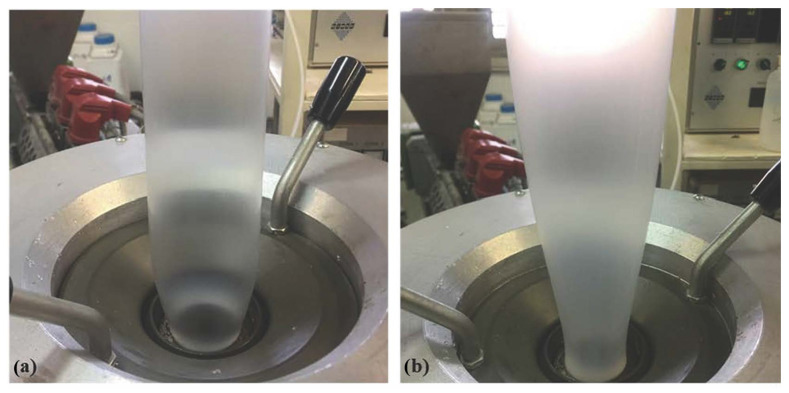
Films of PLA40 produced (**a**) with and (**b**) without bubble cooling.

Through the comparison of the samples produced in the same conditions, but with and without the application of bubble cooling, it can be observed that the absence of cooling air led to significant increase in the TD shrinkage and to a slight decrease in the MD shrinkage. To explain these results, it must be considered that the main TD orientation is given by the bubble expansion that takes place between the die exit and frost line [4,7]. Thus, higher is the frost line height, higher the TD orientation. As evidence of this, it is easy to compare the bubble shapes obtained with and without the use of the cooling air, as reported in Figure 2. The pictures show that films produced with cooling air (Figure 2a) had a lower frost line height, while those obtained without cooling air (Figure 2b) had a significantly higher frost line height. Therefore, with the application of bubble cooling, MD orientation prevails over TD, resulting in films with higher shrinkage in MD than in TD.

In summary, for the samples produced with cooling air and for all the mass flow rates tested, the application of a TUR/BUR ratio higher than 17 for PLA40 and higher than 9 for PLA60 allowed us to maintain an MD shrinkage values between 60% and 80%, which are in typical range of mono-oriented films.

### 3.2. Functional Characterization of the Films

To support the results related to heat shrinking and to better understand the effect of the cooling conditions on the films’ properties, a complete characterization was performed on films PLA40_7 and PLA40_7* and films PLA60_8 and PLA60_8*. These films, according to the samples’ nomenclature, were obtained with the same process conditions except for the cooling air (samples named with the asterisk were produced without cooling air).

#### 3.2.1. Thermal ANALYSIS

**Figure 3:**
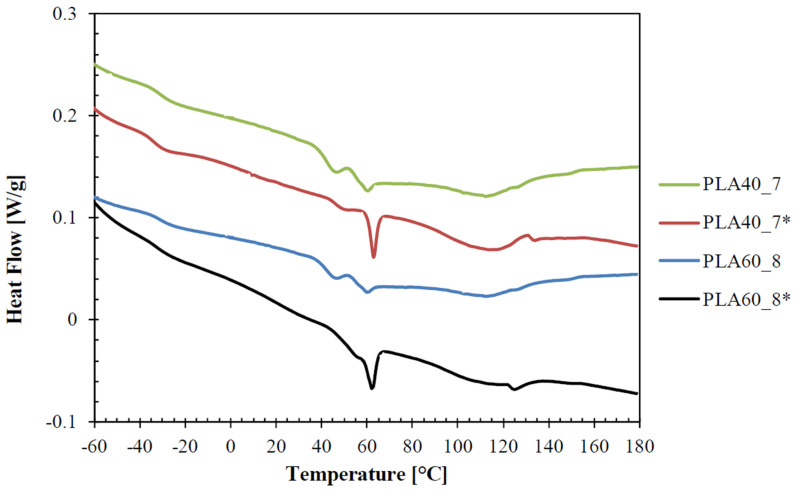
DSC thermograms of the films related to the first heating scan (samples named with the asterisk were produced without cooling air).

Thermograms and the main thermal parameters, such as the glass transition temperature (*T_g_*), the melting temperature (*T_m_*), the melting enthalpy (Δ*H_m_*) of the films are reported in Figure 3 and in Table 5, respectively. They are related to the first heating scan, to investigate the thermal history of the polymers associated with the processing conditions.

The thermograms show the presence of two glass transition temperatures at −33 °C and 60 °C, characteristic of the PBAT and PLA phase, respectively [23]. The presence of both the glass transition temperatures of the polymers is a sign of the immiscibility of PLA and PBAT, which has been extensively reported [30,31,32,33]. For all the films, no melting peaks of PLA can be observed since it is completely amorphous, while PBAT showed two melting peaks. The first narrow and smaller peak (around 45 °C), which was partially overlapped with the PLA glass transition, is related to the butylene-adipate (BA) fraction, whereas the second bigger and broader peak (around 111–114 °C) is related to a common crystal lattice, in which BA units fit into butylene-terephthalate (BT) crystals [34,35,36].

The different PLA amount (40 wt % and 60 wt %) in the two blends did not lead to a significant change in the characteristic transition temperatures of the films. Only a slight decrease in the crystallinity degree of PBAT was observed increasing the PLA content in the blend, which is owing to the effect of PLA that hinders the crystallization of PBAT reducing its chain mobility [17].

The application of cooling air during the film production significantly affected the thermal parameters of the films. In particular, films PLA40_7* and PLA60_8*, which were produced without cooling air, showed a higher glass transition temperature of PLA and a much bigger relaxation peak at 60 °C, compared to films PLA40_7 and PLA60_8, obtained at same drawing conditions, but with cooling air. The increase in the glass transition temperature and in the relaxation enthalpy of PLA can be attributed by a lower mobility of the amorphous chain segments, which have a more rigid and constrained structure [37], therefore suggesting higher orientations of the amorphous PLA segments for the films produced without cooling air (PLA40_7* and PLA60_8*). Since the thermal shrinkage is a measurement of the orientations made in the amorphous fraction [6,38], this outcome is in accordance with the obtained shrinkage values of the films, reported in Table 3 and Table 4, which showed, for PLA40_7* and PLA60_8* films, a significant higher shrinkage in the TD (accompanied only by a slight decrease in the MD) compared to PLA40_7 and PLA60_8 films, respectively. Al-Itry et al. [34] also found that stretching PLA films led to an increase in the glass transition temperature, which was attributable to a more extended and oriented structure of the amorphous phase.

Moreover, thermal results show that films produced without cooling air (PLA40_7* and PLA60_8*), display a reduction in the melting enthalpy of pure BA crystals accompanied by an increase in the second melting enthalpy and temperature. Additionally, it is possible to observe, for the films produced without cooling air, an additional small endothermic peak at higher temperatures, around 125–129 °C, attributable to the presence of a bimodal crystal size and morphology distribution. In particular, since the melting temperature increases with the crystal dimensions [39,40], the lower cooling speed could have led to a bigger size of the PBAT crystals. This is in accordance with other studies [41,42], which reported that a slower cooling rate in the production process provide bigger crystals in polymeric films. Therefore, absence of cooling air could have led to changes in the PBAT crystals morphology and to an increase in their dimensions.

#### 3.2.2. Optical Properties

**Figure 4:**
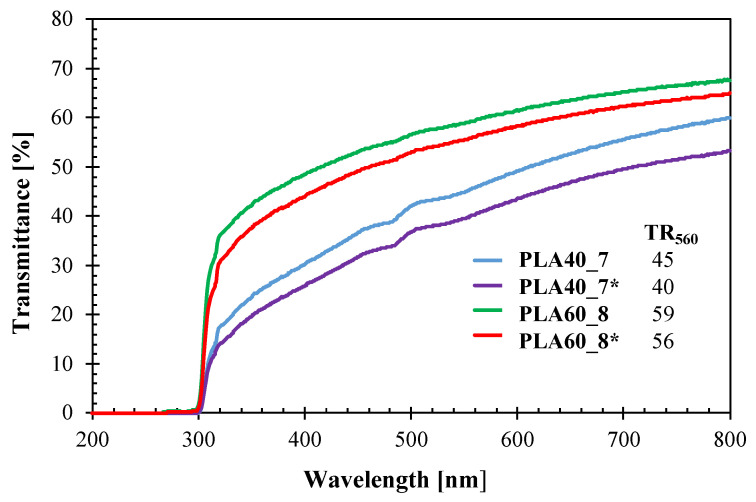
UV–Vis transmittance spectra and percent transparency values at 560 nm (*TR*_560_) of the films (samples named with the asterisk were produced without cooling air).

The whole transmittance spectra in the UV–Vis region and the percent transparency values at 560 nm for the films produced with and without bubble cooling are shown in Figure 4.

The results show that the optical properties of PLA/PBAT films were affected by both the blending ratio and the application of cooling air. Films with a higher content of PLA, corresponding to the PLA60_8 and PLA60_8* samples, had a higher transmittance in the whole analyzed range compared to those with a higher content of PBAT. In fact, since PBAT is opaque while amorphous PLA is transparent [23,38], the increase in PLA content in the blend lead to an increase in the transparency of the films.

Moreover, the absence of cooling air in the film blowing process had a negative impact on the optical properties. According to the previous assumptions on the crystals’ morphology, films produced without cooling air (PLA40_7* and PLA60_8*) exhibit lower transparency compared to their respective films produced with cooling air (PLA40_7 and PLA60_8). Since the crystallinity degree of PBAT was slightly affected by the application of cooling air, the decrease in transparency can be related to the bigger size of the crystals, as a further confirmation of what supposed in previous analyses. Similar results were reported by others [41]; in fact, the higher cooling rates during the extrusion blowing process decreases the haze and increases the glossy of PE films, because of the smaller crystal dimension.

Furthermore, it is worth noting that for all the blends compositions and cooling conditions, the transmittance in the UV wavelength range (λ < 280 nm) of the films was equal to zero (Figure 2). The UV-screening ability is a desirable property especially for the food packaging applications since the UV-light is the responsible of many degradation reactions [43] and shrink films are widely employed in the food packaging field [1].

#### 3.2.3. Mechanical Properties

**Figure 5:**
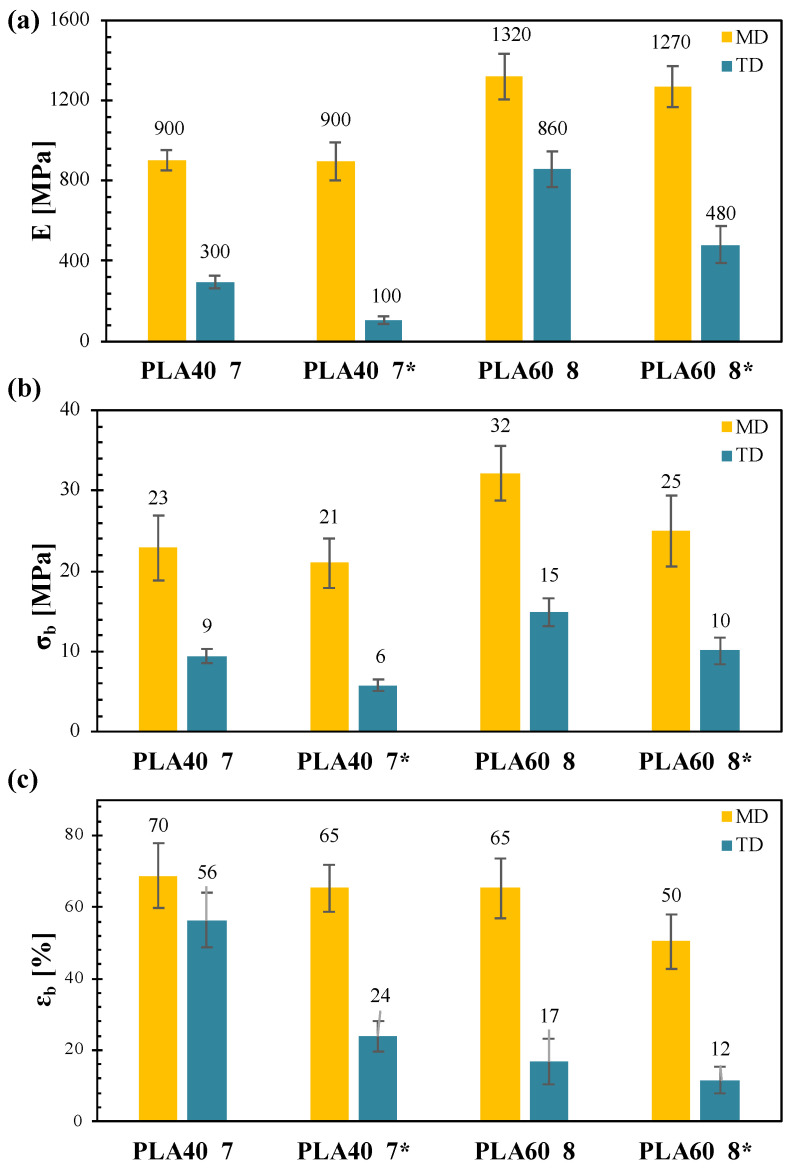
Tensile parameters of the films in MD and TD: (**a**) elastic modulus; (**b**) stress at break; (**c**) elongation at break (samples named with the asterisk were produced without cooling air).

The films were tested for their tensile properties in the machine direction (MD) and in the transverse direction (TD). As an example, Figure 5 compares the main MD and TD tensile parameters (elastic modulus, stress at break and elongation at break) of the films PLA40_7, PLA40_7*, PLA60_8 and PLA60_8*, obtained with the same process conditions (except for the cooling air) and differing in terms of blends composition.

The graphs clearly show that, at fixed processing conditions, the increase in the PLA content in the blend resulted in an increase in the elastic modulus and the stress at break and in a decrease in the elongation at break, as extensively reported in the literature [17,23,44]. Moreover, all the films showed, for each blend composition and cooling speed, better mechanical performances in MD than TD, likely due to the high TUR used that forced the macromolecules to orient mainly in the MD direction.

Another difference in the tensile properties of the films was related to the cooling conditions. The absence of cooling air in the production process, as for PLA40_7* and PLA60_8* films, led to a worsening of the mechanical properties, especially in the TD, even though a higher level of orientation of the amorphous fraction in the TD was reported for these films.

Similar results were reported for polypropylene (PP) films produced with cast film process [45], in which the worsening of the mechanical performance of the samples produced without cooling air was linked to a different morphology of the crystalline phase. In fact, it is known that the presence of few big crystals, instead of many smaller ones, leads to lower density of tie chains in the polymer resulting in a decrease in the mechanical performance of the final system [46]. Therefore, the worse mechanical performance of the films produced without cooling air could be attributable to an increase in PBAT crystals dimensions, in agreement with the thermal and optical analyses.

## 4. Conclusions

Mono-oriented thin shrink films made by blends of amorphous PLA and semi-crystalline PBAT (at 60/40 and 40/60 mass ratio) compatibilized with a multifunctional epoxide chain extender were produced using a film blowing plant varying the TUR/BUR ratio, the mass flow rate, and the cooling air. Shrinkage tests revealed that the produced films have MD shrinkage in the range 60–80% and TD shrinkage in the range 10–20%, which are in the typical range of mono-oriented films. The increase in the TUR/BUR ratio and in the mass flow rate in the film blowing process led to an increase in the longitudinal stretching of the macromolecules with consequent increase in the MD shrinkage of the films. The application of cooling air in the production process was beneficial to obtain mono-oriented shrink films, because it led to a reduction in the TD shrinkage accompanied by a slight enhancement of the MD shrinkage. Moreover, it also led to an improvement of the optical and mechanical properties of the films, attributable to a change in the morphology and to a decrease in the size of the PBAT crystals. The PLA content in the blend also affected the thermal shrinkage and the physical-chemical properties of the films. In particular, at the same process conditions, increasing the percentage of PLA in the blend resulted in higher transparency, stiffness, and shrinkage and in lower ductility of the films. In conclusion, films made by compatibilized blends of PLA and PBAT at 40/60 and 60/40 mass ratios, produced with cooling air and with a TUR/BUR ratio higher than 17 and 9, respectively, showed the best performance as mono-oriented shrink films for packaging applications.

## Figures and Tables

**Table 1:** Films produced from the blend PLA/PBAT 40/60 + 1 wt % Joncryl with their respective process conditions. Films with an asterisk are those produced without cooling air.

Sample Name	Mass Flow Rate (g/min)	Cooling Air	Thickness (μm)	BUR	TUR	TUR/BUR
PLA40_1	9	yes	26	1.9	16	8
PLA40_1*	9	no	26	1.9	16	8
PLA40_2	9	yes	15	1.8	30	17
PLA40_2*	9	no	15	1.8	30	17
PLA40_3	9	yes	14	1.3	44	34
PLA40_4	18	yes	31	1.8	14	8
PLA40_5	18	yes	20	1.7	23	14
PLA40_6	18	yes	16	1.8	29	16
PLA40_7	18	yes	15	1.5	37	25
PLA40_7*	18	no	15	1.5	37	25
PLA40_8	18	yes	10	1.7	47	28
PLA40_8*	18	no	10	1.7	47	28
PLA40_9	36	yes	20	1.8	22	12
PLA40_10	36	yes	15	1.8	31	17
PLA40_11	36	yes	13	1.8	34	19

**Table 2:** Films produced from the blend PLA/PBAT 60/40 + 1 wt % Joncryl with their respective process conditions. Films with an asterisk are those produced without cooling air.

Sample Name	Mass Flow Rate (g/min)	Cooling Air	Thickness (μm)	BUR	TUR	TUR/BUR
PLA60_1	12	yes	50	1.3	12	9
PLA60_2	12	yes	33	1.2	21	18
PLA60_2*	12	no	33	1.2	21	18
PLA60_3	12	yes	20	1.2	32	27
PLA60_4	24	yes	50	1.5	10	7
PLA60_5	24	yes	31	1.5	17	11
PLA60_6	24	yes	28	1.4	20	14
PLA60_7	24	yes	19	1.6	28	18
PLA60_7*	24	no	19	1.6	28	18
PLA60_8	24	yes	15	1.6	34	21
PLA60_8*	24	no	15	1.6	34	21
PLA60_9	48	yes	24	1.9	18	9
PLA60_10	48	yes	20	1.8	22	12
PLA60_11	48	yes	20	1.7	23	14

**Table 3:** Extrusion blowing parameters and thermal shrinkage for films made of PLA40 blend. Films with an asterisk are those produced without cooling air.

Sample Name	Cooling Air	Mass Flow Rate (g/min)	TUR/BUR	MD Shrinkage (%)	TD Shrinkage (%)
PLA40_1	yes	9	8	35 ± 5	11 ± 3
PLA40_1*	no	9	8	33 ± 4	22 ± 3
PLA40_2	yes	9	17	61 ± 4	9 ± 1
PLA40_2*	no	9	17	55 ± 4	11 ± 3
PLA40_3	yes	9	34	74 ± 3	10 ± 2
PLA40_4	yes	18	8	36 ± 3	25 ± 4
PLA40_5	yes	18	14	51 ± 3	26 ± 3
PLA40_6	yes	18	16	65 ± 7	15 ± 4
PLA40_7	yes	18	25	76 ± 5	6 ± 3
PLA40_7*	no	18	25	73 ± 3	18 ± 2
PLA40_8	yes	18	28	79 ± 4	9 ± 1
PLA40_8*	no	18	28	77 ± 6	13 ± 2
PLA40_9	yes	36	12	66 ± 7	11 ± 3
PLA40_10	yes	36	17	75 ± 3	8 ± 1
PLA40_11	yes	36	19	78 ± 5	21 ± 4

**Table 4:** Extrusion blowing parameters and thermal shrinkage for films made of PLA60 blend. Films with an asterisk are those produced without cooling air.

Sample Name	Cooling Air	Mass Flow Rate (g/min)	TUR/BUR	MD Shrinkage (%)	TD Shrinkage (%)
PLA60_1	yes	12	9	46 ± 5	0 ± 1
PLA60_2	yes	12	18	72 ± 5	2 ± 1
PLA60_2*	no	12	18	69 ± 3	7 ± 2
PLA60_3	yes	12	27	80 ± 3	1 ± 1
PLA60_4	yes	24	7	53 ± 4	15 ± 3
PLA60_5	yes	24	11	63 ± 4	10 ± 2
PLA60_6	yes	24	14	72 ± 2	10 ± 3
PLA60_7	yes	24	18	78 ± 2	15 ± 4
PLA60_7*	no	24	18	76 ± 2	19 ± 6
PLA60_8	yes	24	21	81 ± 4	14 ± 6
PLA60_8*	no	24	21	79 ± 2	25 ± 4
PLA60_9	yes	48	9	60 ± 1	9 ± 1
PLA60_10	yes	48	12	75 ± 4	11 ± 3
PLA60_11	yes	48	14	83 ± 5	21 ± 5

**Table 5:** Thermal parameters of the films during the first heating scan (samples named with the asterisk were produced without cooling air).

Sample Name	*T_g_* PBAT (°C)	*T_g_* PLA (°C)	*T_m_*_1_ PBAT (°C)	Δ*H_m_*_1_ PBAT (J/g)	*T_m_*_2_ PBAT (°C)	Δ;*H_m_*_2_ PBAT (J/g)	*X_c_* PBAT (%)
PLA40_7	−33.4	56.1	41.9	1.1	111.4	5.1	9.1
PLA40_7*	−33.1	63.9	49.4	0.3	114.0	6.2	9.5
PLA60_8	−31.7	54.7	45.2	0.9	111.8	3.0	8.5
PLA60_8*	−33.0	60.1	50.5	0.1	113.9	4.0	9.0

## Data Availability

The data presented in this study are available on request from the corresponding author. The data are not publicly available due to privacy restrictions.
